# A fully human connective tissue growth factor blocking monoclonal antibody ameliorates experimental rheumatoid arthritis through inhibiting angiogenesis

**DOI:** 10.1186/s12896-023-00776-8

**Published:** 2023-03-03

**Authors:** Yang Qin, Gan Wu, Jiayi Jin, Hao Wang, Jiani Zhang, Li Liu, Heping Zhao, Jianguang Wang, Xinyu Yang

**Affiliations:** 1 Department of Biochemistry, School of Basic Medical Sciences, Wenzhou Medical University, 325035 Wenzhou, China; 2 Department of Anesthesia and Critical Care, the Second Affiliated Hospital and Yuying, Children’s Hospital of Wenzhou Medical University, Wenzhou, China; 3 Department of Medicinal Chemistry, School of Pharmaceutical Sciences, Wenzhou Medical University, 325035 Wenzhou, China

**Keywords:** CTGF, Phage display, Affinity maturation, Angiogenesis, Arthritis, Human antibody

## Abstract

**Background:**

Connective tissue growth factor (CTGF) plays a pivotal role in the pathogenesis of rheumatoid arthritis (RA) by facilitating angiogenesis and is a promising therapeutic target for RA treatment. Herein, we generated a fully human CTGF blocking monoclonal antibody (mAb) through phage display technology.

**Results:**

A single-chain fragment variable (scFv) with a high affinity to human CTGF was isolated through screening a fully human phage display library. We carried out affinity maturation to elevate its affinity for CTGF and reconstructed it into a full-length IgG1 format for further optimization. Surface plasmon resonance (SPR) data showed that full-length antibody IgG mut-B2 bound to CTGF with a dissociation constant (KD) as low as 0.782 nM. In the collagen-induced arthritis (CIA) mice, IgG mut-B2 alleviated arthritis and decreased the level of pro-inflammatory cytokines in a dose-dependent manner. Furthermore, we confirmed that the TSP-1 domain of CTGF is essential for the interaction. Additionally, the results of Transwell assays, tube formation experiments, and chorioallantoic membrane (CAM) assays showed that IgG mut-B2 could effectively inhibit angiogenesis.

**Conclusion:**

The fully human mAb that antagonizes CTGF could effectively alleviate arthritis in CIA mice, and its mechanism is tightly associated with the TSP-1 domain of CTGF.

**Supplementary Information:**

The online version contains supplementary material available at 10.1186/s12896-023-00776-8.

## Introduction

Rheumatoid arthritis (RA), a common chronic inflammatory autoimmune disorder that mainly affects the joints, is characterized by progressive synovitis, cartilage damage, and bone erosion [1, 2]. As a typical sign of the inflamed rheumatoid synovium, the pannus comprises excessively of proliferated fibroblast-like synoviocytes (FLS), proliferating vessels, and invasive inflammatory cells [3–6]. Notably, angiogenesis accelerates the infiltration of inflammatory cells and proliferation of FLS, and directly leads to pannus formation and destroys adjacent joints [7, 8]. Hence, exploiting novel strategies to inhibit angiogenesis might be the future direction for RA treatment [9, 10].

Recent studies have gradually recognized that connective tissue growth factor (CTGF) plays a critical role in many pathogenic events of RA [11–15]. CTGF, also known as cellular communication network family member 2 (CCN2), is a cysteine-rich protein with four functional domains, including insulin-like growth factor binding protein (IGFBP), thrombospondin type 1 repeat (TSP-1), von Willebrand factor type C repeat (VWC), and C-terminal cystine-knot (CT) modules [14]. CTGF expression was initially observed in endothelial cells and fibroblasts, involved in connective tissue regeneration and angiogenesis [15]. In our previous proteomic study, CTGF was observed substantially upregulated in inflammatory synovium tissue of RA patients and showed a strong connection with cell proliferation and migration [12]. Research conducted by Nozawa et al. has provided evidence that blockade of the CTGF pathway using a mouse monoclonal antibody can ameliorate collagen-induced arthritis (CIA) [13]. Our latest study has shown that TSP-1 is the essential domain for CTGF to enhance angiogenesis, which plays a vital role in forming and maintaining pannus in RA [11]. These results allowed CTGF to be a new antagonistic target, and anti-CTGF antibody may become an attractive option for RA treatment.

Notably, in our multicenter validation cohort study, serum CTGF was an excellent diagnostic biomarker to predict RA, with sensitivity, specificity, positive likelihood, the negative likelihood, positive predictive value and negative predictive value of 0.82, 0.91, 5.74, 0.12, 0.85 and 0.90, respectively [16]. It seemed that more accurate diagnostic capacity with the combination of CTGF and anti-citrullinated protein antibody (ACPA) (AUC = 0.96) than with either ACPA or rheumatoid factor (RF) solely (AUC = 0.80 or 0.79, respectively) [16]. Beyond that, as a diagnostic index of RA, serum CTGF is superior in distinguishing RA from other autoimmune diseases [16]. Therefore, the development of antibodies specifically antagonistic to CTGF is of great significance in diagnosing and treating RA.

Past decades have witnessed that biological agents for the treatment of RA have been gradually introduced into the clinic and appear to have ushered in a groundbreaking era of targeted therapy for RA [17]. Monoclonal antibody (mAb) drugs have gained success in sustaining remission and reducing disease activity. Adalimumab, for example, its global sales reached 20.696 billion dollars in 2021, showing a great application and economical prospect. For a sizable subset of patients, the application of blocking mAbs showed excellent results in alleviating the symptoms and alleviating the progression of RA [18, 19]. Notwithstanding biological agents combined with traditional disease-modifying antirheumatic drugs (DMARDs) therapy could improve the disease control of RA to a certain extent, no more than 20% of patients can get deep remission [20]. Therefore, there is an urgent need to discover more specific disease-related antigens and develop corresponding biological agents.

There has never been a report of a fully human CTGF blocking antibody that can ameliorate arthritis, let alone valuation of its effect. This study analyzed the feasibility of screening a fully human monoclonal antibody with a high affinity for human CTGF (hCTGF) and confirmed its function of ameliorating arthritis in CIA mice. We then verified the role of mAb in inhibiting angiogenesis through in vitro and in vivo experiments. We prospect that this work could contribute new strategies for treating rheumatoid arthritis and developing specific biological agents.

## Results

### Screening and identification of connective tissue growth factor (CTGF) blocking single chain fragment variables (scFvs)

**Fig. 1:**
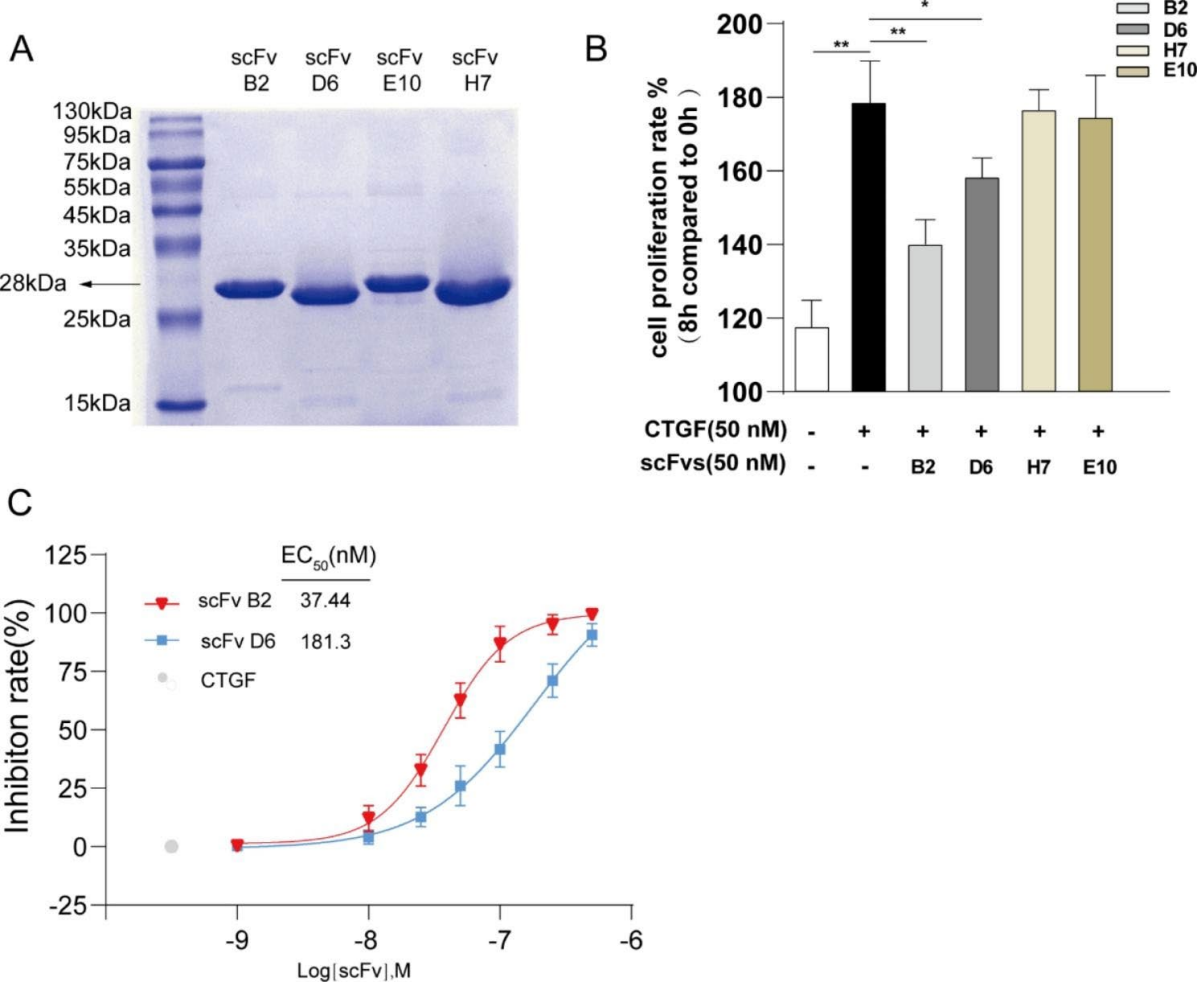
Screening and identification of connective tissue growth factor (CTGF) blocking single chain fragment variables (scFvs). (**A**) SDS-PAGE analysis of purified anti-CTGF scFvs with Coomassie blue staining. (**B**) Cell proliferation rate of HUVECs was evaluated by CCK-8 assay after being treated with recombinant human CTGF (50 nM) and purified anti-CTGF scFvs (50 nM) for 8 h. HUVECs untreated (White bar) and HUVECs only treated with CTGF (Black bar) were used as controls. Formula used to calculate the cell proliferation rate is shown in Methods. (**C**) Inhibition rate of CTGF-induced cell proliferation. Cell viability of HUVECs was evaluated by CCK-8 assay after being treated with recombinant human CTGF (50 nM) and scFv B2 or scFv D6 in increasing concentrations (1-500 nM). HUVECs only treated with CTGF (Grey dot) were used as control. Formula used to calculate the inhibition rate is shown in Methods

Single chain fragment variables (scFvs), the combination of heavy chains (V_H_) and light chains (V_L_) of antibodies connected by flexible linker, retain the ability of antigen binding and are widely used in phage display technology. To screen scFvs with high affinity to human CTGF, a fully human scFv library of 2.72 × 10^9^ complexity was constructed from peripheral blood mononuclear cells (PBMCs) of 50 rheumatoid arthritis patients. After three rounds of affinity biopanning, the number of eluted phages binding to hCTGF increased 914-fold, which were clearly enriched (Additional file 1: Fig. S1A). We prepared 96 clones and screened clones by monoclonal phage ELISA, then four high affinity clones named scFv B2, D6, E10, H7 were selected (absorbance 450 − 650 nm > 1.5) (Additional file 1: Fig. S1B) and expressed for the following research (Fig. 1A). Given that CTGF has the function of enhancing human umbilical vein endothelial cells (HUVECs) cell proliferation dose-dependently [12, 15, 21], we evaluated the capacity of scFvs to antagonize CTGF by a CCK-8 assay. As shown in Fig. 1B, CTGF elevated cell viability of HUVECs by 1.8-fold in 8 h compared to 0 h (p < 0.001). Conversely, this effect was markedly attenuated by 58% and 30% when cells were preincubated with scFv B2 (p < 0.001) and scFv D6 (p < 0.005) at a concentration of 50 nM, respectively. Following this, we chose scFv B2 and scFv D6 for a more comprehensive study. In intervention experiment of gradient concentrations (1-500 nM), as shown in Fig. 1C, scFv B2 showed the more potent anti-CTGF activity with a median effect concentration (EC_50_) of 37.44 nM and scFv D6 with an EC_50_ of 181.3 nM.

### The affinity of anti-CTGF scFv B2 increased after mutagenesis and reconstruction to the IgG1 format

**Fig. 2:**
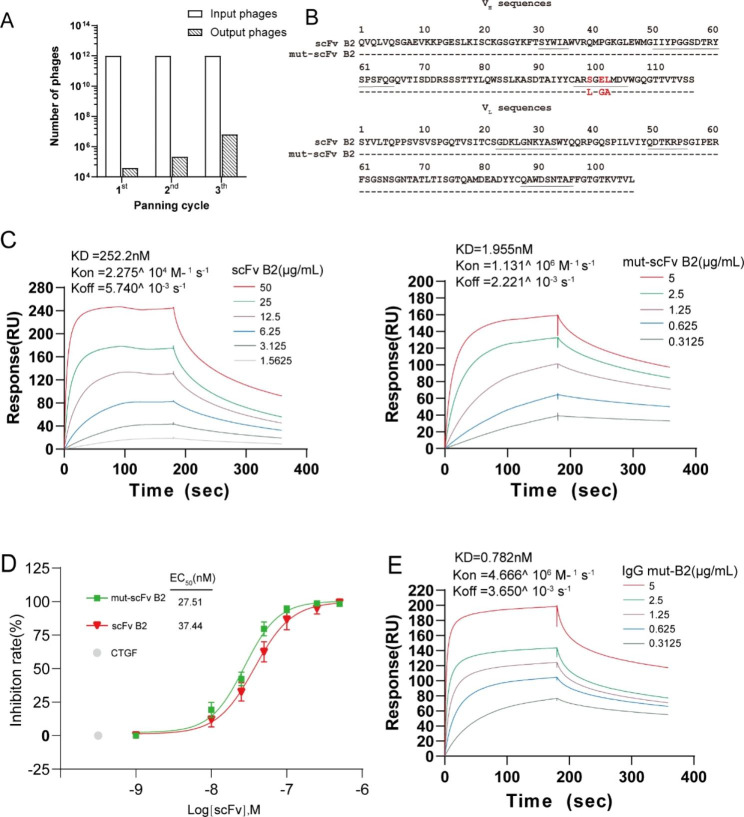
The affinity of anti-CTGF scFv B2 increased after mutagenesis and reconstruction to the IgG1 format. (**A**) The number of eluted phages. After three rounds of screening for CTGF in phage-displayed scFv library with scFv B2-V_H_-CDR3 mutagenesis, the number of eluted phages increased 163-fold over that of the first round. (**B**) Amino acids of the heavy and light chain variable region of scFv B2 and mut-scFv B2. The red letters indicate residues that vary between scFv B2 and mut-scFv B2. Underline: CDR1, CDR2, CDR3 regions of heavy and light chain. (**C**) Affinity of scFv B2 and mut-scFv B2 determined by surface plasmon resonance (SPR). (**D**) Inhibition rate of CTGF-induced cell proliferation. Cell viability of HUVECs was evaluated by CCK-8 assay after treated with recombinant human CTGF (50 nM) and scFv B2 or mut-scFv B2 (1-500 nM) for 8 h. HUVECs only treated with CTGF (white dot) were used as control. Formula used to calculate the inhibition rate is shown in Methods. (**E**) Affinity of IgG mut-B2 determined by SPR.

In general, binding affinity of antibodies is crucial for their application [22]. Although anti-CTGF scFv B2 exhibited the highest binding and antagonistic activity among the four identified scFvs, in order to further improve its binding affinity to better meet the needs of clinical diagnosis and treatment, we adopted phage display-based affinity maturation. We constructed a mutant library by introducing mutants at the hot-spot motifs (RGYW and AGY) in V_H_-CDR3 of scFv B2. After three rounds of affinity biopanning in this mutant library, the number of eluted phages binding to hCTGF increased 163-fold (Fig. 2A). A clone with the highest affinity (mut-scFv B2) was identified. V_H_-CDR3 region of mut-scFv B2 differed from its parental scFv on three amino acid residues: S 99 L; E 101 G; L 102 A (Fig. 2B). The dissociation constant (KD) value of mut-scFv B2 was 1.955 nM while scFv B2’s was 252.2 nM, indicating that the affinity of mut-scFv B2 was 129-fold higher than its parental scFv B2 (Fig. 2C). The parental scFv B2 exhibited a slow association rate (*k*_on_, 2.275 × 10^4^ M^− 1^ s^− 1^) and a dissociation rate (*k*_off_, 5.740 × 10^− 3^ s^− 1^), while after affinity maturation, mut-scFv B2 displayed a dramatically faster association rate (*k*_on_, 1.131 × 10^6^ M^− 1^ s^− 1^) and a moderate slower dissociation rate (*k*_off_, 2.221 × 10^− 3^ s^− 1^) (Fig. 2C). Simultaneously, as shown in Fig. 2D, the antagonistic activity of mut-scFv B2 was also higher than parental scFv B2 (EC_50_ 27.51 nM compared to 37.44 nM). It is known that the serum half-time of scFv format is short (only about 3.5 h) and thus deeply limits its clinical utility [23]. We reconstructed mut-scFv B2 to a human immunoglobulin G1 (IgG1) format, which was a typical strategy to elevate the stability of antibody [24–26], and named it IgG mut-B2 (Additional file 1: Fig. S2). The affinity of mut-scFv B2 (KD = 1.955 nM) increased by 2.5 times after being constructed to full-length IgG1 (KD = 0.782 nM) (Fig. 2E). Interestingly, IgG mut-B2 displayed a moderate higher association rate (*k*_on_, 4.666 × 10^6^ M^− 1^ s^− 1^) while a slightly faster dissociation rate (*k*_off_, 3.650 × 10^− 3^ s^− 1^) than mut-scFv B2. (Fig. 2E)

### Anti-CTGF IgG mut-B2 ameliorated progression of arthritis in a collagen-induced arthritis (CIA) model

**Fig. 3:**
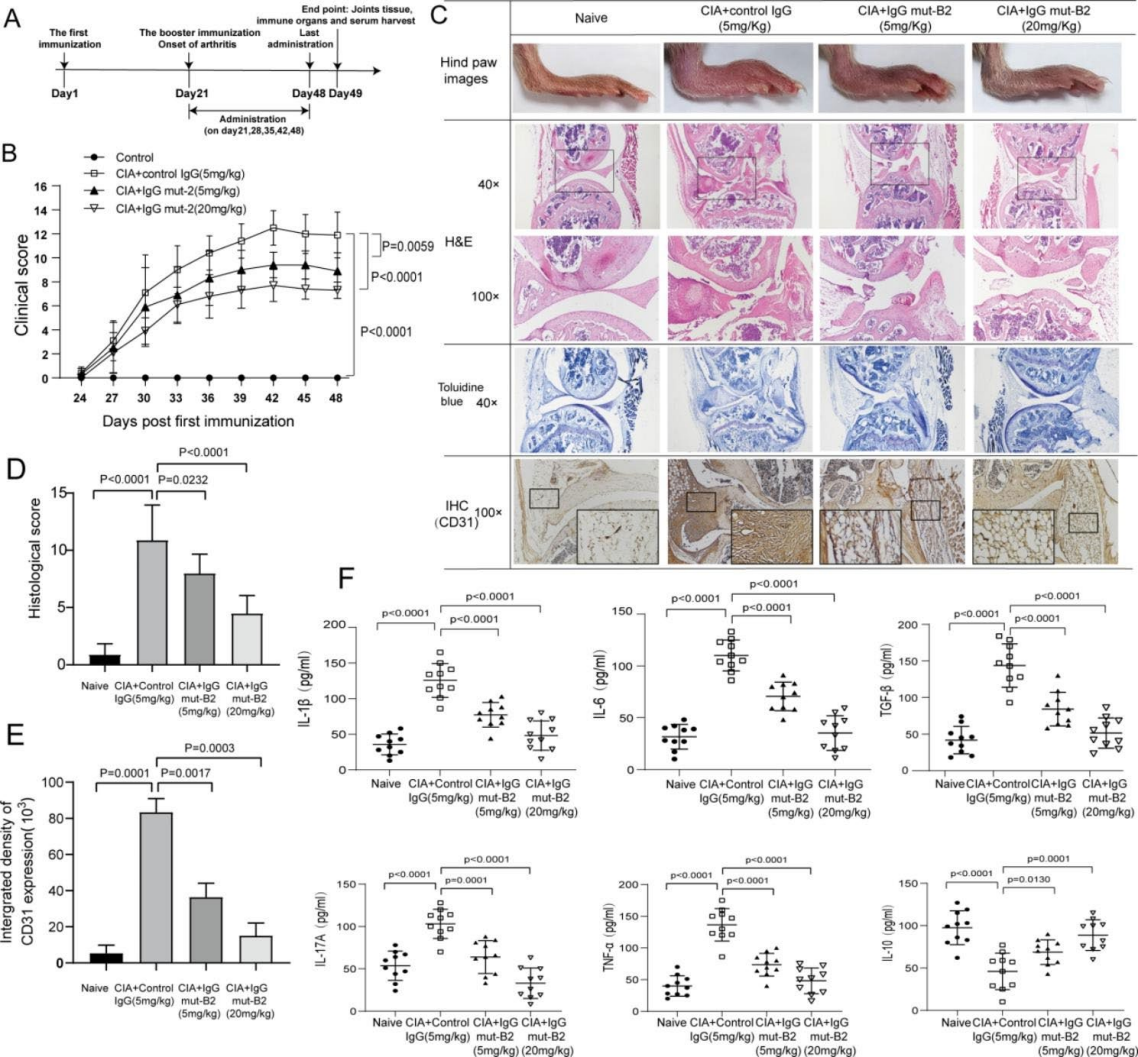
Anti-CTGF IgG mut-B2 ameliorated progression of arthritis in a collagen-induced arthritis (CIA) model. (**A**) Timeline of anti-CTGF IgG mut-B2 and control IgG intervention in CIA mice. (n = 10 per group). (**B**) Clinical scores of CIA mice treated with control IgG or IgG mut-B2. Two independent observers who were unaware of the mice’s treatment examined their arthritis severity every 3 days. The scores were determined as follows: 0: no erythema or swelling, 1: erythema and mild swelling limited to the tarsals or ankle joint, 2: erythema and mild swelling extending from the ankle to the tarsals, 3: erythema and moderate swelling extending from the ankle to the metatarsal joints, 4: erythema and severe swelling encompassing the ankle, paw, and digits, or ankylosis of the limb. Statistical significance was determined by analysis of variance (ANOVA) of repeated measurements. (n = 10 per group). (**C**) Articular tissues of CIA mice were stained by H&E and toluidine blue. Expression of CD31 in paraffin sections of synovium samples of CIA mice was determined by IHC analysis. (**D**) Histological scores of CIA mice based on analysis of synovial hyperplasia, cartilage and bone erosion, and inflammatory cell infiltration shown in (C). (**E**) Intergrated density of CD31 expression in synovial tissues of CIA mice. (**F**) Concentrations of IL-1β, IL-6, TNF-α, IL-17 A, IL-10 in serum of CIA mice were detected by ELISA. (n = 10 per group). All reactions were conducted in triplicate and data were presented with mean ± SD.

To verify the in vivo function produced by IgG mut-B2 through blocking CTGF, we used a CIA mouse model, one of the most classic RA models, to study the efficacy of IgG mut-B2 in the progress of RA, as illustrated in Fig. 3A. Clinical scores and paw swelling were significantly reduced after injecting IgG mut-B2, compared with CIA mice injected with control IgG (Fig. 3B, Additional file 2: Table S3). Meanwhile, IgG mut-B2 intervention reduced the histological scores of CIA mice (Fig. 3C, D). In addition, IgG mut-B2 intervention decreased immunohistochemical CD31 positivity in the synovial tissue which means a decrease of blood vessels (Fig. 3C, E). Moreover, IgG mut-B2 intervention down-regulated the circulating levels of pro-inflammatory cytokines TNF-α, IL-6, IL-1β, IL-17 A, TGF-β, and upregulated the circulating level of anti-inflammatory cytokine IL-10 in CIA mice (Fig. 3F).

### The TSP-1 domain of CTGF is essential for the interaction between anti-CTGF IgG mut-B2 and CTGF

**Fig. 4:**
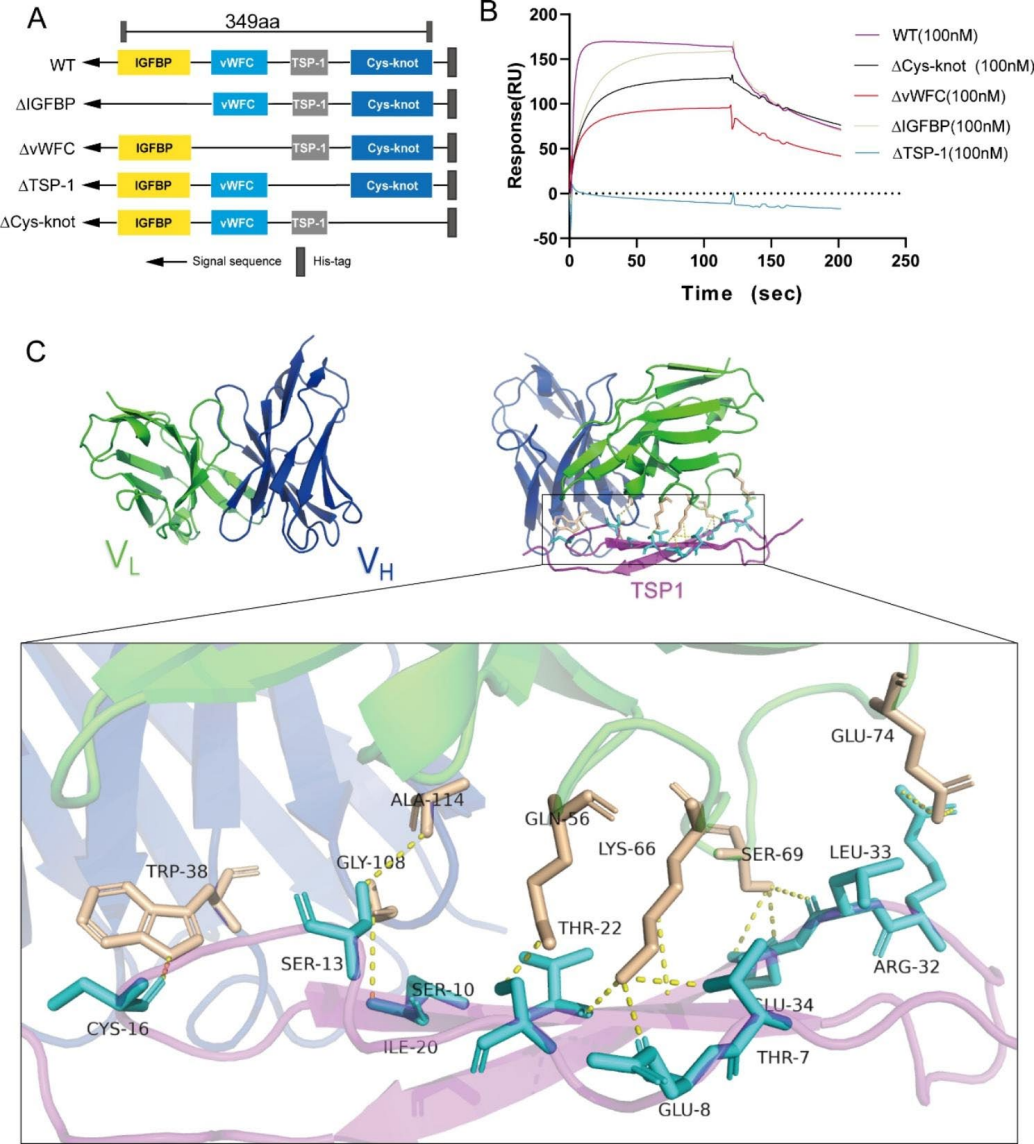
IgG mut-B2 interacted with CTGF through TSP-1 domain. (**A**) Schematic of full-length CTGF (WT) and its deletion mutants. (**B**) Binding affinity of IgG mut-B2 to CTGF and its deletion mutants determined by SPR assay. (**C**) Molecular modeling of variable regions of IgG mut-B2 and TSP-1 domain of CTGF. Left panel: Three-dimensional model of variable regions of IgG mut-B2 structure predicted by homology modeling. The heavy chains’ variable regions are shown in blue and the light chains’ variable regions in green. Right panel: The intermolecular interaction analyses of the IgG mut-B2 with TSP-1 domain. TSP-1 domain of CTGF is represented by magenta. The sticks model is used to demonstrate the key residues involved in the interactions, and the key residues of TSP-1 domain are marked in cyan, key residues of antibody are marked in light orange. Yellow dotted lines represent hydrogen bonds

We then aimed to determine which structural domain was essential for the interaction between IgG mut-B2 and CTGF. The design of domain mapping was shown in Fig. 4A. Through surface plasmon resonance technology (SPR), we demonstrated that IgG mut-B2 interacted with WT CTGF and truncation mutants unless the TSP-1 domain of CTGF was deleted (Fig. 4B). Additionally, the epitope-paratope interaction details for the high affinity of IgG mut-B2 to TSP-1 domain were detected by computer-based homology modeling. The three-dimensional (3D) structures of Fv regions of IgG mut-B2 were shown in Fig. 4C, and then IgG mut-B2 were successively docked with TSP-1 domain (Fig. 4C). By comparative analysis on these two complexes, it was found that the interaction residues of IgG mut-B2 including Trp38, Gly108, Ala114 in V_H_, and Gln56, Lys66, Ser69, Glu74 in V_L_ form 13 hydrogen bonds against Thr7, Glu8, Ser10, Ser13, Cys16, Ile20, Thr22, Arg32, Leu33 and Glu34 in TSP-1 domain, which means IgG mut-B2 interacted with TSP-1 domain with high affinity. The findings above indicated that IgG mut-B2 interacts with CTGF via the TSP-1 domain.

### Effects of anti-CTGF IgG mut-B2 on angiogenesis

**Fig. 5:**
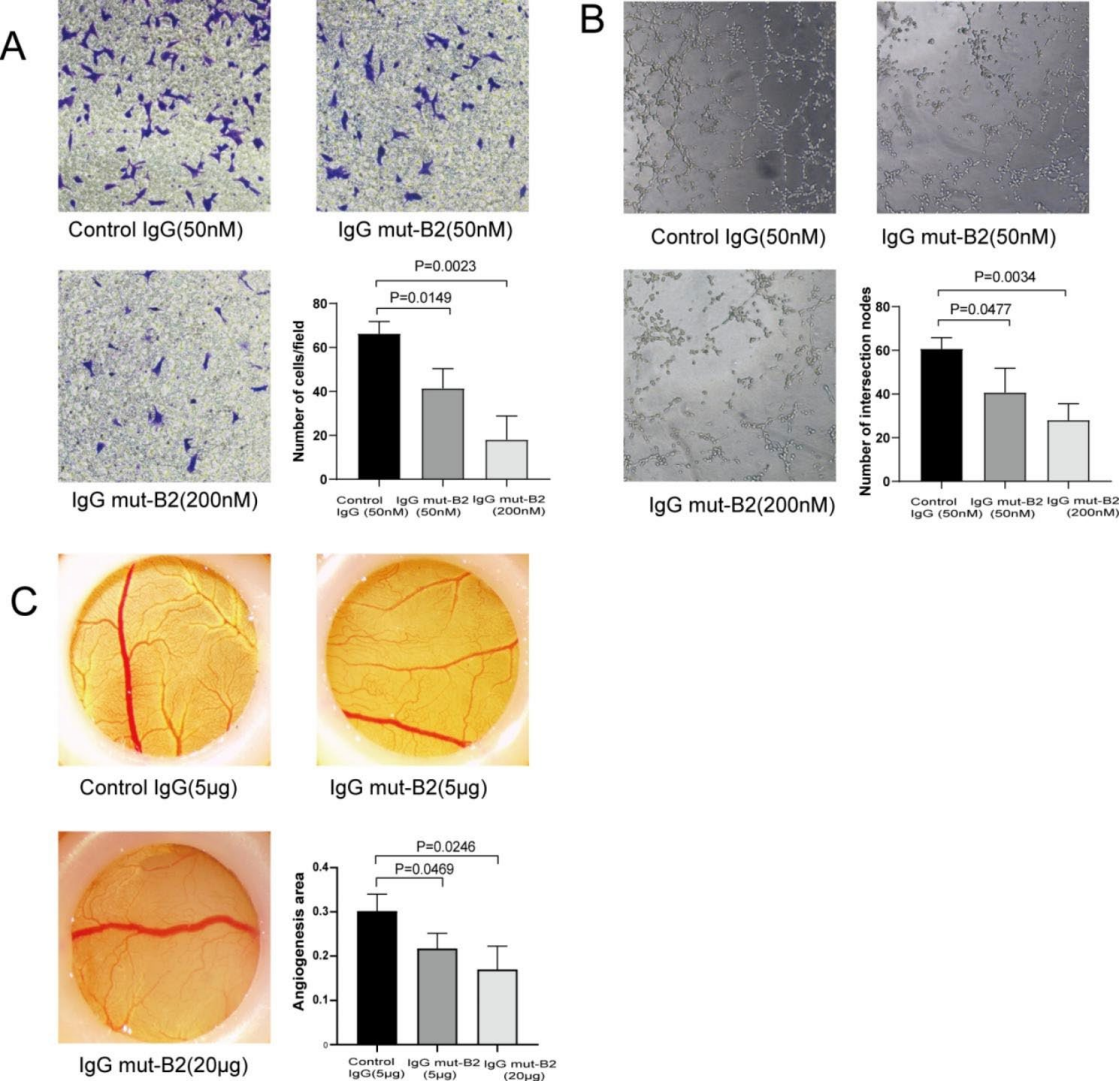
Effect of IgG mut-B2 on angiogenesis. (**A**) Cell migration assay used to assess angiogenesis in vitro. Lower right panel: numbers of HUVEC per field. (Original magnifcation, ×200) (**B**) Tube formation assay used to assess angiogenesis in vitro. Lower right panel: numbers of intersection nodes. (Original magnifcation, ×100) (**C**) Chorioallantoic membrane (CAM) assay used to assess angiogenesis in vivo. Lower right panel: ratio of vascular area to CAM area

We previously reported that CTGF relied on the TSP-1 domain to enhance angiogenesis [11], which plays a key role in pannus formation and maintenance in RA. Therefore, we investigated if IgG mut-B2 inhibits angiogenesis by binding to the TSP-1 domain. For this purpose, in vitro, HUVECs used for Transwell assays and tube formation experiments were treated with control IgG or IgG mut-B2 at 37 °C. The Transwell assay result showed that IgG mut-B2 inhibited the migration ability of HUVECs in a dose-dependent manner (Fig. 5A). The tube formation experiments showed a similar result to the Transwell assays (Fig. 5B). The findings above in vitro allowed us to test the ability of IgG mut-B2 to prevent angiogenesis in vivo through chorioallantoic membrane (CAM) assays. Adding IgG mut-B2 to CAM inhibited angiogenesis in chicken embryos to a great extent, the formation of small vessels decreased compared with control (Fig. 5C). Based on these experiments, we confirmed that IgG mut-B2 has an efficient anti-angiogenesis function and thereby alleviates the progression of RA.

## Discussion

In recent years, the application of biological agents, such as Adalimumab (TNF-α inhibitor), has shown obvious advantages in alleviating the progression of RA [17]. However, about 30% RA patients remain insensitive to biological agents due to the limited drug targets [27–29]. Therefore, the drug development of new target is urgently needed. CTGF is considered as a hub gene in RA [30], which promotes aggressive pannus formation resulting in bone destruction, cartilage injury and inflammatory factor release [7, 8]. CTGF is an ideal target of drug intervention in RA.

Therefore, we worked on developing a treatment strategy that targets CTGF in this study. Initially, we built a fully human scFv library from PBMCs of RA patients. On the one hand, screening in a scFv library from human instead of immunized mice, could effectively avoid the drawbacks of subsequent optimization such as antibody humanization, which could elicit human anti-mouse antibody responses (HAMA) that diminish mAb efficacy [31]. On the other hand, due to the high expression of CTGF in RA, the scFv library from RA patients may contain a higher abundance of anti-hCTGF antibodies. Compared with screening a healthy human scFv library with high background and low abundance of anti-hCTGF antibodies, this strategy can significantly reduce the screening difficulty [32–35]. We then successfully generated a fully human, potent scFv by screening this library with hCTGF. Following this, we carried out affinity maturation for further optimization. The SPR analysis showed it was a successful strategy and the antibody affinity increased 163-fold from 252.2 nM to 1.955 nM.

The main disadvantages of scFv are its poor stability, ease of accumulation and precipitation. Due to small molecular weight, scFv is easy to be cleared by the kidney (half-time is about 3.5 h) [23]. Those drawbacks also affect the therapeutic effect of long retention. Reconstruction to full-length IgG was a typical strategy to elevate scFv stability [24–26]. Although IgG2 and IgG4 Fc fragments can be employed to generate full-length antibodies when Fc effector function is undesirable for therapeutic efficacy, they have worse conformational stability than IgG1 due to their proclivity to aggregate [36, 37]. Furthermore, disulfide-bond reshuffling and Fab-arm exchange in the IgG2 and IgG4 subclasses result in several heterogeneous isoforms with different activity [38, 39]. Therefore, in this study, we used an IgG1 Fc fragment to generate full-length anti-CTGF antibody. Reconstruction to full-length IgG1 format also slightly improved antibody affinity from 1.955 nM to 0.782 nM.

We then verified the in vivo anti-arthritis potency of IgG mut-B2 in CIA mice. As one of the most classic and reliable models to test a therapeutic intervention, angiogenesis and synovium inflammation develop parallel during the progression of CIA, which highly mimic the progression of angiogenesis in RA [40]. IgG mut-B2 showed great capacity to reduce pathological scores, paw swelling and synovial hyperplasia in a dose-dependent manner. The Immunohistochemistry of CD31 also showed decreased synovial vessels in CIA mouse after intervention.

We further explore the direct combination pattern between IgG mut-B2 and CTGF. The distinctive structure of CTGF confers its ability to participate in various biological processes, and the TSP-1 domain was reported to be tightly associated with cell adhesion and tissue repairing [41]. Our previous study demonstrated that CTGF interacts with ANXA2 protein through the TSP-1 domain to enhance angiogenesis [11]. Through SPR and molecular docking, we verified that IgG mut-B2 interacted with CTGF through the TSP-1 domain. Furthermore, the following experiments demonstrated that IgG mut-B2 has a superior capacity to prevent angiogenesis both in vitro and in vivo.

In conclusion, we successfully generated a novel, fully human, anti-CTGF monoclonal antibody by phage display technology. Our data showed a great function of anti-CTGF mAb in ameliorating arthritis by suppressing angiogenesis. Our research reveals the feasibility of CTGF as a therapeutic target and the effectiveness of anti-CTGF mAb as therapeutic agents, which helps to bring new insights to exploit therapeutic strategies for RA and once again emphasize the importance of CTGF in the pathogenesis of RA.

## Conclusion

Taken together, CTGF is a promising therapeutic target, in this study, we generated a fully new human CTGF blocking antibody through phage display technology and this antibody showed a great function in ameliorating arthritis. Besides, it showed an effective function in inhibiting angiogenesis, and this mechanism was tightly associated with the TSP-1 domain of CTGF.

## Materials and Methods

### Patient samples

We obtained peripheral blood samples from 50 RA patients who fulfilled the 2010 American College of Rheumatology (ACR) criteria at the First Affiliated Hospital of Wenzhou Medical University (Wenzhou, China). This study was approved by the local research ethics committee and samples were extracted with the informed consent of patients.

### Construction of scFvs library

Total RNA of peripheral blood mononuclear cells (PBMCs) from 50 RA patients were extracted using trizol reagent (T9108, Takara) and retrotranscribed. The variable region genes of the heavy (V_H_) and light chain (V_L_) of the synthesized cDNA were subsequently amplified through PCR in condition as follows: 95 °C for 30 s, 55 °C for 15 s, and 72 °C for 30 s, for a total of 35 cycles. The primers used to amplify the variable genes are shown in the Additional file 2: Table S1. V_H_ or V_L_ DNA of different patients were recovered and mixed randomly. After that, scFv DNA was amplified through overlapping extension PCR, in which the mixed V_H_ and V_L_ DNA were used as templates and the classical (Gly_4_Ser)_3_ sequence was used as a linker. The second round of PCR reaction settings were as follows: 95 °C for 30 s, 60 °C for 15 s, 72 °C for 30 s, for 30 cycles. The second round of PCR product was used as templates for the third round of PCR. The third round PCR reaction conditions were as follows: 95 °C for 30 s, 60 °C for 30 s, 72 °C for 1 min, for 30 cycles. Primer used for the second, third round of PCR are shown in the Additional file 2: Table S1. The recovered V_H_-Linker-V_κ_ and V_H_-Linker-V_λ_ DNA were mixed and then inserted to phagemid pCANTAB5E. To produce phages, *E. coli* TG1 electroporated with the phagemids were cultured in 2YTAG medium (100 µg/mL of ampicillin and 1% of glucose), added with M13KO7 (N0315S, NEB), the helper phage at an MOI ratio of 20 at 37℃ for 30 min. After centrifugation, *E. coli* TG1 cells were resuspended and shook overnight at 30℃. The phages in the supernatant were then precipitated by the addition of PEG/NaCl. The scFv library size was estimated by the standard plate colony counting method.

### Affinity screening of anti-CTGF scFvs

The phage library (0.1 mL) was blocked with PBS (0.5 mL) containing 3% (w/v) milk and incubated with 50 µL streptavidin magnetic beads (21344, ThermoFisher Scientific) for 2 h. Meanwhile, 10 µg biotinylated hCTGF (9190-CC, R&D) was incubated with another blocked 50 µL beads for 1 h, after three times of wash, the blocked phage supernatant was incubated with CTGF-binding beads for 1 h. The phage-antigen beads mixture was washed with 0.1% PBST for 15 times (0.3%, 0.5% for the second and third screening, respectively), phages that specifically bound to CTGF were eluted with 400 µL Gly-HCl solution (pH = 3) and subsequently neutralized with 200 µL Tris-HCl solution (pH = 9). The recovery was amplified by infecting *E. coli* TG1 for the second and third rounds of screening. Phage amplification was carried out previously described [34]. Titers of eluted phages in every round were calculated to confirm the enrichment after screening. Briefly, the eluted products were diluted 10^3^, 10^4^, 10^5^, 10^6^ times respectively and then used to infect *E. coli* TG1. The infected *E. coli* TG1 could grow on the ampicillin plate with 2YT medium, and the titer was calculated according to the colony number on the plate.

### Monoclonal phage ELISA

Streptavidin matrix coated 96-Well Plates (22351, Beaver bio) was coated with 10 µg biotinylated CTGF in coating buffer (PBS) for 1 h and then blocked. *E. coli* TG1 was infected with the eluted phages of the third screening and grew on ampicillin plate overnight, ninety-six clones were selected and cultured in a 96-well deep plate for 16 h. After that, the 96-well deep plate was centrifuged at 8000 g, the pellets were resuspended and cultured at 37℃ for 30 min in 2YTAG medium (100 µg/mL of ampicillin and 1% of glucose), added with M13KO7, then centrifuged again, resuspended and shook overnight at 30℃ to produce phages. To determine phage affinity property, 200 µL supernatant containing phages of ninety-six clones were collected and incubated with the prepared plate for 1 h at 37℃, then washed and incubated with anti-M13 Antibody, HRP-conjugated (11973-MM05T-H, Sino biological). 100 µL TMB Substrate Solution (SEKCR01, Sino biological) was added for color reaction of 10 min and 2 M H_2_SO_4_ was added to stop the reaction. The absorbance was read at the 450 nm wavelength using a spectrophotometer.

### Expression and purification of anti-CTGF scFvs

The genes of scFvs showing high specific affinity for CTGF were inserted into the pET28a vector (Novagen). ScFvs with a 6× his-tag at the C terminus were induced for 6 h in bacterial culture by adding 0.5mM IPTG (ST098, Beyotime). After culture, bacteria cells were sonicated and centrifuged. Supernatant containing protein was incubated with Ni Sepharose (17-5318-06, GE Healthcare) in 4℃ for column purification. After incubation for 1 h, Sepharose was washed with Tris-HCl solution (pH = 7.4) containing 5 mM imidazole three times and bound protein was eluted with Tris-HCl solution (pH = 7.4) containing 0.5 M imidazole. Purified scFvs were completely dialyzed with PBS, and analyzed by reducing SDS-PAGE. SDS-PAGE was stained with coomassie blue staining solution (P0017F, Beyotime) for 30 min, and washed with ddH_2_O overnight in a shaker for analysis. The SDS-PAGE was imaged with the GelDoc XR + IMAGELAB (Bio-Rad).

### Cell proliferation assay

The viability of human umbilical vein endothelial cells (HUVECs) in 96-well plate was measured by CCK-8 assay system (CK04, Dojindo). Briefly, HUVECs were plated on 96-well plate (5 × 10^3^ per well), treated with CTGF (50 nM) and with or without anti-CTGF scFvs (1-500 nM). After 8 h intervention, 10 µL CCK-8 solution was added to each well and incubated for 1 h in 37 ℃. The absorbance was measured at 450 nm. The cell proliferation rate was calculated using the following formula: [Absorbance (8 h) – Absorbance (control)] / [Absorbance (0 h) – Absorbance (control)] × 100%, absorbance of well that only contains culture medium and CCK-8 kit was used as Absorbance (control). Inhibition rate was calculated using the following formula: [(cell proliferation rate of only CTGF-treated HUVECs - cell proliferation rate of scFv-treated HUVECs) / (cell proliferation rate of CTGF-treated HUVECs - cell proliferation rate of untreated HUVECs)] ×100%. The experiments were repeated in triplicate.

### Affinity maturation of mut-scfv B2

Affinity maturation was carried out as previously described [42, 43]. Briefly, a phage-displayed scFv library containing the V_L_ and V_H_ gene repertoire of scFv B2 was constructed, with random mutations introduced at amino acid residues of V_H_-CDR3. Primers designed to introduce random mutations to the hotspots (RGYW and AGY) of V_H_-CDR3 are shown in the Table 1. The screening process was described as previous, after 3 rounds of in vitro screening, a clone with the highest affinity (mut-scFv B2) was identified and expressed.

**Table 1:** Primers for mutation of scFv B2 V_H_-CDR3

Sense	5’-ATATATTACTGTGCGAGANNSGGGNNSNNSATGGACGTCTGGGGCCAGGG-3’
Anti-sense	5’- CGCACAGTAATATATGGCGGTGTCCGAGGCCTTCAGGCTGCTCCACT-3’

NNS codon (N randomizing with all four nucleotides and S introducing only C or G)

### Expression and purification of full-length antibody and CTGF truncation mutants

For expression of full-length antibody, V_H_ and V_L_ of mut-scFv B2 were respectively reconstructed into pcDNA3.1 with antibody constant area, the sequences of full length IgG1 format are shown in Additional file 2: Table S2. Then the plasmids were transiently expressed with ExpiCHO™ expression system (A29133, ThermoFisher Scientific). After culture for 9 days, antibody in supernatant was harvested and purified by Protein G Sepharose (17061801, GE Healthcare) through column purification. Briefly, Protein G Sepharose was incubated with supernatant for 1 h and washed with PBS (pH = 7.4). After that, antibody binding to Sepharose was eluted by Gly-HCl solution (pH = 2.9) and subsequently neutralized with Tris-HCl solution (pH = 9). Protein concentration was calculated after dialysis with PBS.

For expression of CTGF truncation mutants, plasmids were transiently expressed with ExpiCHO™ expression system. After culture for 9 days, protein in supernatant was harvested and purified with Ni Sepharose (17371201, GE Healthcare) through column purification. Briefly, Ni Sepharose was incubated with supernatant for 1 h and washed with PBS (pH = 7.4). After that, protein was eluted by Tris-HCl solution (pH = 7.4) containing 0.5 M imidazole. Protein concentration was calculated after dialysis with PBS. Plasmids for expression of CTGF truncation mutants were constructed as previously described [11].

### Surface plasmon resonance (SPR) assay

The binding properties of scFvs or IgG were analyzed using a Biacore T200 instrument (GE Healthcare). Firstly, CTGF was coupled on the surface of CM5 chip (BR100530, GE Healthcare) through standard amine coupling, then, scFvs or IgG were diluted with running buffer PBS (pH = 7.4) to corresponding concentrations, filtrated with a 0.22 μm filter (SLGP033N, Millipore), and pumped over the chip surface for 3 min at a flow rate of 10 µl/min, in the order of descending concentration. The pure running buffer was added for another 5 min to continue the dissociation followed by NaCl injection (1 M) for 30 s at 30 µl/min to achieve regeneration. The binding of CTGF truncation mutants to IgG was detected using the same instrument with an CM5 chip coupled with IgG. The data collected were evaluated using a Biacore T200 Evaluation software version 3.2.1.

### Collagen-induced arthritis (CIA) model

DBA/1 mice (male, 8 weeks, 18–20 g) were purchased from Shanghai SLAC Laboratory and reared in a Specific pathogen-free room at Laboratory Animal Centre of Wenzhou Medical University. Mice were randomized into four groups of ten mice in this experiment. On day 1, mice were given 100 µL of type II bovine collagen (2 mg/mL) (20021, Chondrex) emulsified in equal amounts of Freund’s complete adjuvant (7009, Chondrex) intradermally at the base of the tail. On day 21, the mice received a booster injection of 100 µL of type II bovine collagen (2 mg/mL) emulsified in equal volumes of Freund’s incomplete adjuvant (7002, Chondrex). Mice were subsequently given intraperitoneally IgG mut-B2 or control IgG every seven days until day 48 and sacrificed on day 49. The mice under deep anesthesia were euthanized through spine dislocation after intraperitoneal injection of pentobarbital. Serum and joint tissues were taken for further investigation. The Institutional Animal Care and Use Committee of Wenzhou Medical University authorized all techniques in the animal tests, which followed UK (Home Office) regulations. The study was carried out in compliance with the ARRIVE guidelines.

### Histological analysis

The knee joints were fixed, decalcified, embedded, and sectioned in 5-µm intervals for staining. H&E staining was carried out as previously described [44], toluidine blue staining was carried out using a topical application of 1% toluidine blue solution (G3668, Solarbio) for 10 min and destaining with acetic acid (1%) for 20 s. For immunohistochemical analysis, a diaminobenzidine kit (P0202, Beyotime) was used. Briefly, sections placed on slides were deparaffinized, rehydrated, antigen-unmasked, permeabilized, blocked, and incubated with a rabbit polyclonal CD31 antibody (ER31219; HuaBio) at a 1:250 dilution. Subsequently, a peroxidase DAB detection system was applied. The sections were observed using a Nikon photomicroscope. Histological scores were assessed as previously described [45].

### ELISA

Circulating levels of IL-6, TNF-α, IL-17 A, IL-1β, TGF-β and IL-10 were detected by ELISA kits (Abcam, USA) according to the manufacturer’s protocols. Following the instructions, all samples were diluted to 100 µL, then incubated with the capture antibody and detection antibody. Plates were read at 450 nm. The experiments were repeated in triplicate.

### Homology modeling and molecular docking

The structure models of antibody Fv regions of IgG mut-B2 were built via homology modeling using the ABodyBuilder server [46]. The structure models of TSP-1 domain were built via homology modeling using Modeller 9.25 program as previously described [11]. IgG mut-B2 and the TSP-1 domain were docked using the Autodock 4.2.6 program. Then, the docked poses with the lowest binding free energies were selected as the best conformations and the key interacting residues in those docked complexes were analyzed and mapped by Pymol software version 2.3.0.

### Transwell and tube formation assays

After being subjected to serum starvation for 24 h, HUVECs (1 × 10^5^ in 300 µL serum-free medium) were plated in the upper chambers of 24-well Transwell with 8.0-µM pore polycarbonate membrane filters (3422, Corning), then 500 µL medium was added to the lower chamber of 24-well Transwell with control IgG (50 nM) or IgG mut-B2 (50, 200 nM). After incubation for 6 h, non-migrating cells were removed and cells that migrated to the underside of the upper chamber were fixed with 4% paraformaldehyde, then stained with crystal violet (G1062; Solarbio). After being washed, chambers were observed and photographed under the microscope. The cell number was measured by IMAGE J software [47]. The experiments were repeated in triplicate.

The tube formation assay was performed as follows: a mixture of matrigel (356234, BD) thawing in 4℃ overnight and DMEM medium at a ratio of 1:1 was added to 96-well plates 70 µL/well and incubated at 37℃ for solidification. HUVECs were resuspended at a concentration of 5 × 10^5^ /ml and put into plates 100 µL/well, treated with control IgG (50 nM) or IgG mut-B2 (50, 200 nM). After incubation of 6 h, tube-like structures were captured by microscope and digital camera, branch points and tubular length were assessed by IMAGE J software. The experiments were repeated in triplicate.

### Chicken embryo allantoic membrane experiment

Fertilized eggs were hatched at 37℃ for 7 days, on the eighth day, alive chicken embryos were checked. Then the egg sheet was gently peeled to expose the air chamber. The allantoic membrane was removed and placed with a silicone ring. Next, eggs were treated with control IgG or IgG mut-B2 and cultured at 37℃. After three days the blood vessels of embryos were captured by microscope and digital camera. The vascular and chorioallantoic membrane regions were assessed using the IMAGE J software. The angiogenic area was calculated as a percentage using the following formula: vascular area/CAM area ×100%. The experiments were repeated in triplicate.

### Statistical analysis

The experimental data were analyzed using SPSS 22.0. Two groups of data that met the normal distribution and homogeneity of variance were analyzed using Student’s unpaired t-test and data that unmet normal distribution and homogeneity of variance were analyzed using the Mann-Whitney U-test. Only p values smaller than 0.05 were statistically significant.

## Electronic supplementary material

Below is the link to the electronic supplementary material.

Supplementary Material 1

Supplementary Material 2

Supplementary Material 3

## Data Availability

The data that support the findings of this study are available from the corresponding author upon reasonable requires.

## Abbreviations

CTGF: connective tissue growth factor
RA: rheumatoid arthritis
mAb: monoclonal antibody
scFv: single-chain fragment variable
SPR: surface plasmon resonance
CIA: collagen-induced arthritis
CAM: chorioallantoic membrane
KD: dissociation constant
FLS: fibroblast-like synoviocytes
IGFBP: insulin-like growth factor binding protein
TSP-1: Thrombospondin type 1 repeat
VWC: Von Willebrand factor type C repeat
CT: C-terminal cystine-knot
ACPA: anti-citrullinated protein antibody
RF: rheumatoid factor
DMARDs: disease-modifying antirheumatic drug
hCTGF: human connective tissue growth factor
ACR: American College of Rheumatology
PBMCs: peripheral blood mononuclear cells
HUVECs: human umbilical vein endothelial cells
EC_50_: median effect concentration
3D: three-dimensional
