# Supplementary material for: A fully human connective tissue growth factor blocking monoclonal antibody ameliorates experimental rheumatoid arthritis through inhibiting angiogenesis
Source: BMC Biotechnol. 2023 Mar 3;23:6. doi: 10.1186/s12896-023-00776-8 (PMC9985226; doi:10.1186/s12896-023-00776-8)
Supplement: Supplementary file 1 — Supplementary Material 1 [file 12896_2023_776_MOESM1_ESM.docx]

**Supplementary materials**

**Supplementary Figure S1.** (**A**) The number of eluted phages. After 3 rounds of screening for hCTGF recombinant protein, the number of eluted phages increased 914‐fold over that of the first round. (**B**) Binding affinity of 96 phage-display clones bind to biotinylated CTGF determined by ELISA.

**
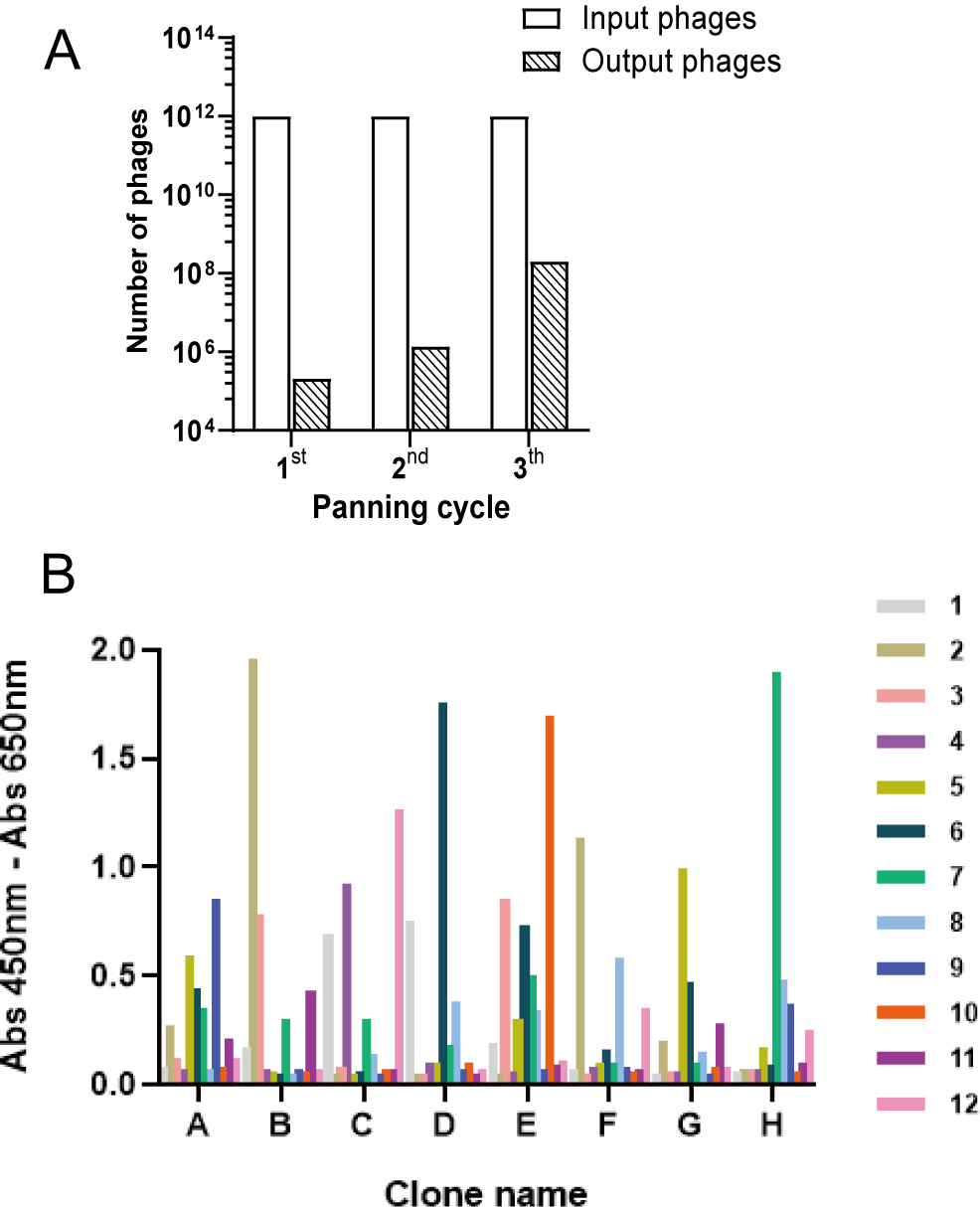
**

**Supplementary Figure S2.** The heavy (55 kDa) and light chains (25 kDa) of purified IgG mut-B2 antibody were analyzed by SDS-PAGE.

**
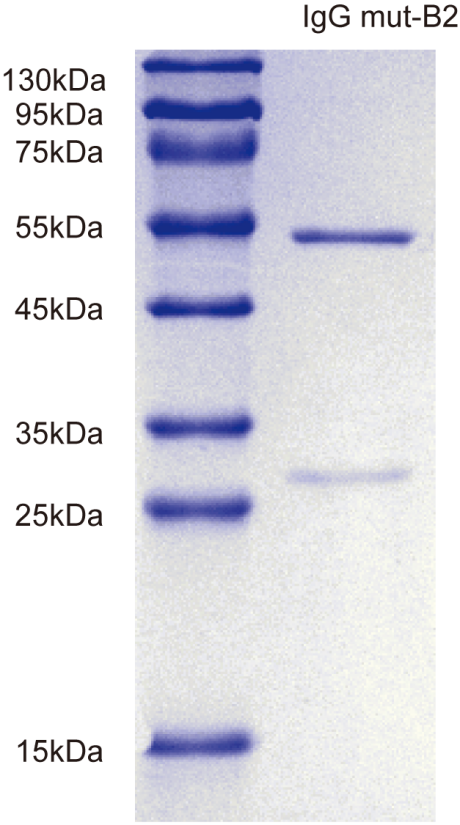
**
