# Supplementary material for: A fully human connective tissue growth factor blocking monoclonal antibody ameliorates experimental rheumatoid arthritis through inhibiting angiogenesis
Source: BMC Biotechnol. 2023 Mar 3;23:6. doi: 10.1186/s12896-023-00776-8 (PMC9985226; doi:10.1186/s12896-023-00776-8)
Supplement: Supplementary file 2 — Supplementary Material 2 [file 12896_2023_776_MOESM2_ESM.docx]

**Supplementary materials**

**Supplementary Table S1.** Primers for PCR amplification of human antibody variable genes.

| **Primers for primary amplifications of V_H_ genes** | |
| --- | --- |
| HuV_H_ 1aBACK | 5’-CAGGTGCAGCTGGTGCAGTCTGG-3’ |
| HuV_H_ 2aBACK | 5’-CAGGTCAACTTAAGGGAGTCTGG-3’ |
| HuV_H_ 3aBACK | 5’-GAGGTGCAGCTGGTGGAGTCTGG-3’ |
| HuV_H_ 4aBACK | 5’-CAGGTGCAGCTGCAGGAGTCGGG-3’ |
| HuV_H_ 5aBACK | 5’-GAGGTGCAGCTGTTGCAGTCTGC-3’ |
| HuV_H_ 6aBACK | 5’-CAGGTACAGCTGCAGCAGTCAGG-3’ |
| HuJ_H_l-2FOR plus | 5’-CGCCTCCACCTGAGGAGACGGTGACCAGGGTGCC-3’ |
| HuJ_H_3FOR plus | 5’-CGCCTCCACCTGAAGAGACGGTGACCAT TGTCCC-3’ |
| HuJ_H_4-5FOR plus | 5’-CGCCTCCACCTGAGGAGACGGTGACCAGGGTTCC-3’ |
| HuJ_H_6FOR plus | 5’-CGCCTCCACCTGAGGAGACGGTGACCGTGGTCCC-3’ |
| **Primers for primary amplifications of Vκ genes** | |
| HuVκ1aBACK plus | 5’-TGGCGGATCGGACATCCAGATGACCCAGTCTCC-3’ |
| HuVκ2aBACK plus | 5’-TGGCGGATCGGATGTTGTGATGACTCAGTCTCC-3’ |
| HuVκ3aBACK plus | 5’-TGGCGGATCGGAAATTGTGTTGACGCAGTCTCC-3’ |
| HuVκ4aBACK plus | 5’-TGGCGGATCGGACATCGTGATGACCCAGTCTCC-3’ |
| HuVκ5aBACK plus | 5’-TGGCGGATCGGAA ACGACACTCACGCAGTCTCC-3’ |
| HuVκ6aBACK plus | 5’-TGGCGGATCGGAAATTGTGCTGACTCAGTCTCC-3’ |
| HuJκ1 FOR | 5’-ACGTTTGATTTCCACCTTGGTCCC-3’ |
| HuJκ2 FOR | 5’-ACGTTTGATCTCCAGCTTGGTCCC-3’ |
| HuJκ3 FOR | 5’-ACGTTTGAT ATCCACTTTGGTCCC-3’ |
| HuJκ4 FOR | 5’-ACGTTTGATCTCCACCTTGGTCCC-3’ |
| HuJκ5 FOR | 5’-ACGTTTAATCTCCAGTCGTGTCCC-3’ |
| **Primers for primary amplifications of Vλ genes** | |
| Huλ1BACK plus | 5’- TGGCGGATCGCAGTCTGTGTTGACGCAGCCGCC-3’ |
| Huλ2BACK plus | 5’- TGGCGGATCGCAGTCTGCCCTGACTCAGCCTGC-3’ |
| Huλ3aBACK plus | 5’- TGGCGGATCGTCCTATGTGCTGACTCAGCCACC-3’ |
| Huλ3bBACK plus | 5’- TGGCGGATCGTCTTCTGAGCTGACTCAGGACCC-3’ |
| HL4back plus | 5’- TGGCGGATCGCAGCCTGTGCTGACTCARYC-3’ |
| Huλ5BACK plus | 5’- TGGCGGATCGCAGGCTGTGCTCACTCAGCCGTC-3’ |
| Huλ6BACK plus | 5’- TGGCGGATCGAATTTTATGCTGACTCAGCCCCA-3’ |
| HuJλ1FOR | 5’-ACCTAGGACGGTGACCTTGGTCCC-3’ |
| HuJλ2-3FOR | 5’-ACCTAGGACGGTCAGCTTGGTCCC-3’ |
| HuJλ7FOR | 5’-GAGGACGGTCAGCTGGGTGC-3’ |
| **Primers for second amplifications of VH genes** | |
| HuV_H_1aBACKSfi | 5’-GTCCTCGCAACTGCGGCCCAGCCGGCCATGGCCCAGGTGCAG CTGGTGCAGTCTGG-3 |
| HuV_H_2aBACKSfi | 5’-GTCCTCGCAACGCGGCCCAGCCGGCCATGGCCCAGGTCAACTTA AGGGAGTCTGG-3’ |
| HuV_H_3aBACKSfi | 5’-GTCCTCGCAACTGCGGCCCAGCCGGCCATGGCCGAGGTGCAGCTGGTGGAGTCTGG-3’ |
| HuV_H_4aBACKSfi | 5’-GTCCTCGCAACTGCGGCCCAGCCGGCCATGGCCCAGGTGCAGCTG CAGGAGTCGGG-3’ |
| HuV_H_5aBACKSfi | 5’-GTCCTCGCAACTGCGGCCCAGCCGGCCATGGCCGAGGTGCAGCTG TTGCAGTCTGC-3’ |
| HuV_H_6aBACKSf | 5’-GTCCTCGCAACTGCGGCCCAGCCGGCCATGGCCCAGGTACAGCTG CAGCAGTCAGG-3’ |
| Linker-r | 5’-CGATCCGCCACCGCCAGAACCACCTCCGCCTGAACCGCCTCCACC-3’ |
| **Primers for second amplifications of Vκ genes** | |
| HuJκ1FORNot | 5’-GAGTCATTCTCGACTTGCGGCCGCACGTTTGATTTCCACCTTGGTCCC-3’ |
| HuJκ2FORNot | 5’-GAGTCATTCTCGACTTGCGGCCGCACGTTTGATCTCCAGCTTGGTCCC-3’ |
| HuJκ3FORNot | 5’-GAGTCATTCTCGACTTGCGGCCGCACGTTTGATATCCACTTTGGTCCC-3’ |
| HuJκ4FORNot | 5’-GAGTCATTCTCGACTTGCGGCCGCACGTTTGATCTCCACCTTGGTCCC-3’ |
| HuJκ5FORNot | 5’-GAGTCATTCTCGACTTGCGGCCGCACGTTTAATCTCCAGTCGTGTCCC-3’ |
| Linker-s | 5’-GGTGGAGGCGGTTCAGGCGGAGGTGGTTCTGGCGGTGGCGGATCG-3’ |
| **Primers for second amplifications of Vλ genes** | |
| HuJλ1FORNot | 5’-GAGTCATTCTCGACTTGCGGCCGCACCTAGGACGGTGACCTTGGTCCC-3’ |
| HuJλ2-3FORNot | 5’-GAGTCATTCTCGACTTGCGGCCGCACCTAGGACGGTCAGCTTGGTCCC-3’ |
| HL7FORNot | 5’-GAGTCATTCTCGACTTGCGGCCGCGAGGACGGTCAGCTGGGTGC-3’ |
| Linker-s | 5’-GGTGGAGGCGGTTCAGGCGGAGGTGGTTCTGGCGGTGGCGGATCG-3’ |
| **Primers for for third amplifications for assembling V_H_-(G4S1)3 linker-VL scFv** | |
| Sfi I | 5’-GTCCTCGCAACTGCGGCCCAGCCGGCCATG GCC-3’ |
| Not I | 5’-GAGTCATTCTCGACTTGCGGCCGC-3’ |

Double underline: linker; Underline: restriction sites.

**Supplementary Table S2.** DNA sequences of IgG1format antibody.

| **Sequences of heavy chain** | |
| --- | --- |
| Signal peptide | 5’-ATGGACATGAGGGTGCCAGCTCAGCTGCTGGGACTGCTGCTGCTGTGGCTGCCTGGAGCGCGCTGT-3’ |
| The variable region gene of the heavy chain (V_H_) | 5’-CAGGTGCAGCTGGTGCAATCTGGGGCAGAGGTGAAAAAGCCCGGGGAGTCTCTGAAGATCTCCTGTAAGGGTTCTGGATACAAGTTTACCAGTTACTGGATCGCCTGGGTGCGCCAGATGCCCGGGAAAGGTCTGGAGTGGATGGGGATCATCTATCCTGGTGGCTCTGATACCAGATACAGCCCGTCCTTCCAAGGCCAGGTCACCATCTCAGACGACAGGTCCAGCAGCACCACCTACCTGCAGTGGAGCAGCCTGAAGGCCTCGGACACCGCCATATATTACTGTGCGAGACTCGGGGGGGCTATGGACGTCTGGGGCCAGGGGACCACGGTCACCGTCTCCTCA-3’ |
| The constant region gene of the heavy chain (C_H_) | 5’-GCGTCGACAAAGGGACCATCCGTGTTCCCACTGGCTCCCAGCTCTAAGTCCACCAGCGGAGGAACAGCCGCTCTGGGCTGTCTGGTGAAGGACTATTTCCCAGAGCCCGTGACCGTGAGCTGGAACTCTGGCGCCCTGACCAGCGGAGTGCATACATTTCCTGCTGTGCTGCAGTCCAGCGGCCTGTACTCTCTGTCTTCCGTGGTGACCGTGCCAAGCTCTTCCCTGGGCACCCAGACATATATCTGCAACGTGAATCACAAGCCAAGCAATACAAAGGTGGATAAGAGGGTGGAGCCCAAGTCTTGTGACAAGACCCATACATGCCCCCCTTGTCCTGCTCCAGAGCTGCTGGGAGGACCATCCGTGTTCCTGTTTCCACCCAAGCCTAAGGATACCCTGATGATCTCCCGGACCCCAGAGGTGACATGCGTGGTGGTGGATGTGAGCCACGAGGACCCCGAGGTGAAGTTTAACTGGTACGTGGACGGCGTGGAGGTGCATAATGCTAAGACCAAGCCTAGGGAGGAGCAGTACAACTCTACCTATCGGGTGGTGTCCGTGCTGACAGTGCTGCACCAGGATTGGCTGAACGGCAAGGAGTATAAGTGCAAGGTGTCTAATAAGGCCCTGCCCGCTCCTATCGAGAAGACCATCTCCAAGGCCAAGGGCCAGCCTAGGGAGCCACAGGTGTACACACTGCCTCCATCTCGGGATGAGCTGACCAAGAACCAGGTGTCCCTGACATGTCTGGTGAAGGGCTTCTATCCCTCCGACATCGCTGTGGAGTGGGAGAGCAATGGCCAGCCTGAGAACAATTACAAGACCACACCCCCTGTGCTGGATTCCGACGGCAGCTTCTTTCTGTATAGCAAGCTGACCGTGGACAAGTCTCGCTGGCAGCAGGGCAACGTGTTTTCTTGTTCCGTGATGCATGAGGCCCTGCACAATCATTACACACAGAAGAGCCTGTCTCTGTCCCCAGGCAAGTAG-3’ |
| **Sequences of light chain** | |
| Signal peptide | 5’-ATGGACATGAGGGTGCCAGCTCAGCTGCTGGGACTGCTGCTGCTGTGGCTGCCTGGAGCGCGCTGT-3’ |
| The variable region gene of the light chain (V_L_) | TCCTATGTGCTGACTCAGCCACCCTCAGTGTCCGTGTCCCCAGGACAGACAGTCAGCATAACCTGCTCTGGAGATAAATTGGGGAATAAATACGCTTCCTGGTATCAGCAGAGGCCAGGCCAGTCCCCTATACTGGTCATCTATCAAGATACCAAGCGGCCCTCAGGGATCCCTGAGCGATTCTCTGGCTCCAACTCTGGGAACACAGCCACTCTGACCATCAGCGGGACCCAGGCTATGGATGAGGCTGACTATTACTGTCAGGCGTGGGACAGCAACACTGCCTTCTTCGGGACTGGGACCAAGTTAACCGTCCTA |
| The constant region gene of the light chain (C_L_) | 5’-GGCCAGCCAAAGGCTGCTCCATCTGTGACCCTGTTCCCACCTTCCAGCGAGGAGCTGCAGGCCAACAAGGCTACCCTGGTGTGCCTGATCTCCGACTTTTACCCAGGAGCTGTGACAGTGGCTTGGAAGGCTGATTCTTCCCCTGTGAAGGCTGGCGTGGAGACCACAACCCCATCTAAGCAGTCCAACAATAAGTACGCCGCCTCCTCTTATCTGAGCCTGACCCCCGAGCAGTGGAAGTCTCACAGGTCCTATAGCTGCCAGGTGACACATGAGGGCTCCACAGTGGAGAAGACCGTGGCCCCTACAGAGTGTAGCTAG-3’ |

**Supplementary Table S3.** Clinical scoring data of CIA mice

| Day | Control | | | | | | | | | | CIA+control IgG(5mg/kg) | | | | | | | | | | CIA+IgG mut-2(5mg/kg) | | | | | | | | | | CIA+IgG mut-2(20mg/kg) | | | | | | | | | |
| --- | --- | --- | --- | --- | --- | --- | --- | --- | --- | --- | --- | --- | --- | --- | --- | --- | --- | --- | --- | --- | --- | --- | --- | --- | --- | --- | --- | --- | --- | --- | --- | --- | --- | --- | --- | --- | --- | --- | --- | --- |
| 24 | 0 | 0 | 0 | 0 | 0 | 0 | 0 | 0 | 0 | 0 | 0 | 0 | 1 | 1 | 1 | 0 | 1 | 0 | 0 | 0 | 0 | 0 | 1 | 0 | 1 | 0 | 1 | 0 | 0 | 0 | 0 | 0 | 0 | 0 | 0 | 0 | 0 | 0 | 0 | 0 |
| 27 | 0 | 0 | 0 | 0 | 0 | 0 | 0 | 0 | 0 | 0 | 1 | 3 | 1 | 4 | 6 | 4 | 3 | 4 | 4 | 1 | 4 | 4 | 1 | 2 | 6 | 0 | 2 | 0 | 5 | 1 | 3 | 2 | 1 | 2 | 6 | 2 | 1 | 0 | 3 | 1 |
| 30 | 0 | 0 | 0 | 0 | 0 | 0 | 0 | 0 | 0 | 0 | 6 | 5 | 8 | 10 | 10 | 7 | 5 | 4 | 13 | 3 | 6 | 6 | 4 | 4 | 10 | 4 | 6 | 4 | 13 | 2 | 4 | 5 | 3 | 4 | 6 | 4 | 3 | 4 | 4 | 2 |
| 33 | 0 | 0 | 0 | 0 | 0 | 0 | 0 | 0 | 0 | 0 | 6 | 8 | 10 | 11 | 11 | 9 | 9 | 7 | 12 | 7 | 6 | 7 | 7 | 6 | 9 | 5 | 7 | 7 | 12 | 3 | 6 | 7 | 7 | 6 | 7 | 4 | 7 | 7 | 7 | 3 |
| 36 | 0 | 0 | 0 | 0 | 0 | 0 | 0 | 0 | 0 | 0 | 9 | 10 | 10 | 12 | 12 | 10 | 9 | 10 | 13 | 9 | 9 | 9 | 6 | 7 | 8 | 5 | 9 | 10 | 13 | 7 | 8 | 7 | 6 | 7 | 9 | 5 | 9 | 7 | 7 | 3 |
| 39 | 0 | 0 | 0 | 0 | 0 | 0 | 0 | 0 | 0 | 0 | 9 | 12 | 12 | 13 | 13 | 11 | 10 | 11 | 13 | 10 | 9 | 10 | 8 | 8 | 9 | 7 | 9 | 8 | 13 | 9 | 8 | 8 | 5 | 7 | 9 | 6 | 9 | 8 | 8 | 5 |
| 42 | 0 | 0 | 0 | 0 | 0 | 0 | 0 | 0 | 0 | 0 | 10 | 13 | 13 | 14 | 14 | 13 | 11 | 12 | 14 | 11 | 10 | 9 | 9 | 9 | 10 | 8 | 9 | 9 | 12 | 9 | 9 | 9 | 5 | 8 | 8 | 8 | 9 | 7 | 8 | 6 |
| 45 | 0 | 0 | 0 | 0 | 0 | 0 | 0 | 0 | 0 | 0 | 11 | 10 | 10 | 14 | 14 | 12 | 11 | 11 | 14 | 13 | 9 | 10 | 8 | 9 | 9 | 10 | 8 | 10 | 12 | 9 | 8 | 8 | 6 | 8 | 7 | 8 | 8 | 8 | 6 | 7 |
| 48 | 0 | 0 | 0 | 0 | 0 | 0 | 0 | 0 | 0 | 0 | 8 | 11 | 11 | 15 | 14 | 11 | 13 | 12 | 12 | 12 | 8 | 9 | 7 | 8 | 10 | 10 | 7 | 9 | 12 | 9 | 8 | 7 | 8 | 7 | 7 | 8 | 8 | 7 | 6 | 7 |
