# Supplementary material for: A fully human connective tissue growth factor blocking monoclonal antibody ameliorates experimental rheumatoid arthritis through inhibiting angiogenesis
Source: BMC Biotechnol. 2023 Mar 3;23:6. doi: 10.1186/s12896-023-00776-8 (PMC9985226; doi:10.1186/s12896-023-00776-8)
Supplement: Supplementary file 3 — Supplementary Material 3 [file 12896_2023_776_MOESM3_ESM.docx]

**Supplementary materials**

**Original picture of Fig. 1D and Supplementary Fig. S2**


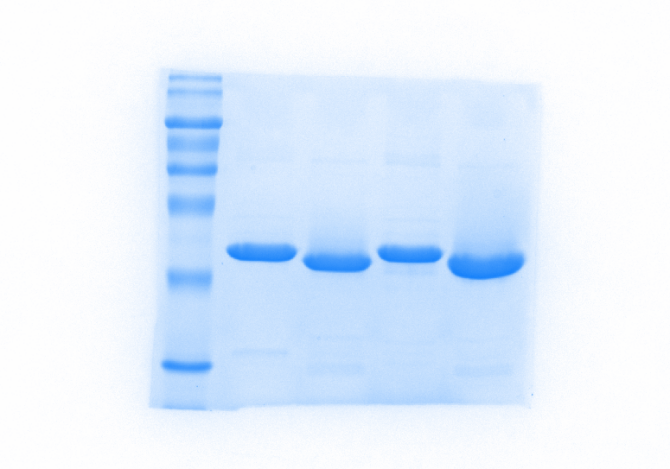


The original gel and blot image of Fig. 1D : SDS-PAGE analysis of purified anti-CTGF scFvs with Coomassie blue staining. Line1: marker, line2-5:scFv B2, scFv D6, scFv E10, scFv H7.


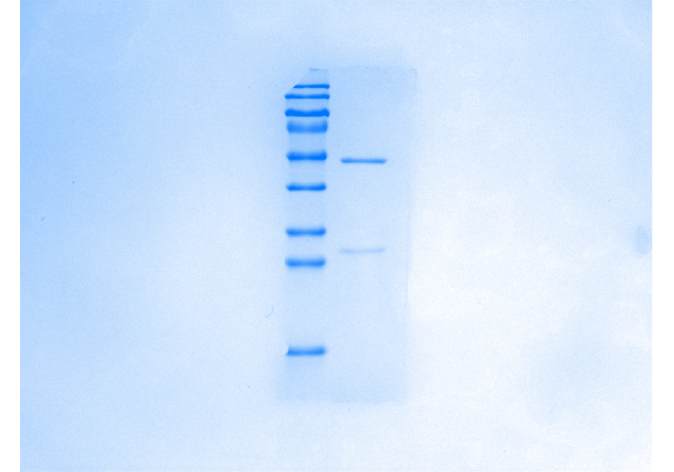


The original gel and blot image of Supplementary Fig. S2 : The heavy (55 kDa) and light chains (25 kDa) of purified IgG mut-B2 antibody were analyzed by SDS-PAGE. Line1: marker, line2:IgG mut-B2.
